# Pulsatile high-dose treatment with antiangiogenic tyrosine kinase inhibitors improves clinical antitumor activity

**DOI:** 10.1007/s10456-017-9555-8

**Published:** 2017-04-18

**Authors:** Maria Rovithi, Henk M. W. Verheul

**Affiliations:** 1 Department of Medical Oncology, Cancer Center Amsterdam, VU University Medical Center, De Boelelaan 1117, Room 3A46, 1081 HV Amsterdam, Noord-Holland The Netherlands; 2 Medical Oncology Unit, Department of Internal Medicine, Agios Nikolaos General Hospital, Agios Nikolaos, Crete, Greece

Angiogenesis comprises one of the core hallmarks of cancer, essential for tumor establishment and growth. Elucidating the components of the angiogenic molecular pathway led to the identification of the crucial signaling axis of vascular endothelial growth factor (VEGF). This facilitated the discovery and subsequent clinical integration of either monoclonal antibodies targeting the soluble form of VEGF, such as bevacizumab, or small molecules that block the intracellular kinase domain of VEGF receptor, thereby preventing downstream signaling. More specifically, the development of small molecule tyrosine kinase inhibitors (TKIs) was initially accompanied by two hypotheses, tightly intertwined; they exert their action exclusively on endothelial cells, not tumor cells, and consequently, since endothelial cells are genetically stable, these drugs are “resistant to resistance,” namely their efficacy is not limited by the development of resistance [1].

The drug development of antiangiogenic TKIs is mainly based on the concept that inhibition of angiogenesis induces tumor cell death indirectly, through substrate deprivation. Treatment strategies with TKIs are traditionally focused on continuous administration, some with built-in intervals to allow recovery from toxicities. The subsequent plateau in drug plasma concentration was hypothesized to induce continuous inhibition of angiogenesis via blockade of specific intracellular signaling. Proof of concept for this dogma is lacking, because it is currently impossible to determine inhibitory activity of these drugs in individual cells. In other words, no adequate and validated technique is available to assess drug-related inhibition of specific signaling cascades in the targeted endothelial cells in tumor samples from patients during treatment.

TKIs are small, hydrophobic molecules that can easily cross the cellular membrane, enter the cell and interact with intracellular kinases. Their blocking capacity is dependent on their affinity for individual kinases [2]. Therefore, one may wonder how they could discriminate between different cell types and target one exclusively. In contrast, they most likely affect not only endothelial cells, but may direct their inhibitory effects on tumor cells and cells of the tumor microenvironment as well. In all these cells, the inhibitory activity of (antiangiogenic) TKIs is dependent on their discriminative affinity for specific kinases, the intracellular concentrations reached and the presence and activity of kinases in these cells. Based on some of these considerations, we studied the effect of multiple TKIs in vitro and found that they exhibit a direct tumoricidal effect on cancer cell lines in clinically relevant concentrations [3], as those measured in on-treatment tumor biopsies from mice and patients (Labots et al., unpublished data). Disassociating antiangiogenic TKIs from the concept of antiangiogenesis-only mediating function, these agents can potently inhibit tumor growth both in vitro and in vivo.

Clinical application of treatment with antiangiogenic TKIs showed in multiple tumor types their potential to improve patient survival but simultaneously exposed their limitations. Failing expectations, intrinsic or, more commonly, acquired resistance remains a major hurdle and accounts for heterogeneous patient response to treatment [4].

In most cases, acquired resistance becomes apparent after a prolonged treatment period of almost daily treatment and thereby continuous exposure. Simulation of this clinical setting has been conducted in the laboratory setting. By continuous exposure for months of cancer cell lines to increasing TKI concentrations, a resistant phenotype could be established. Crucial characteristics of this acquired resistance pattern included the increased intracellular drug accumulation accompanied by an increase in the lysosomal storage capacity and the reversibility of the resistance with restitution of sensitivity upon removal of the drug from the culture medium [5]. The cytocidal effect of TKIs is reportedly apoptosis-mediated, while autophagy acts as an adaptive mechanism to treatment, offering a further argument in support of the pivotal role of tumor cell lysosomes in the cellular adaptations upon treatment with these drugs [6].

Based on these findings and alternative mechanisms of action that can be anticipated from treatment with antiangiogenic TKIs, further optimization of their clinical use is urgently needed in order to fully exploit their potential antitumor activity. Coming from the concept of daily continuous dosing to inhibit angiogenesis to a more tumor cell directed approach by changing schedule and dosing, we hypothesized that short exposure to high concentration of TKIs may improve their antitumor activity. This hypothesis is supported by several published data on dose escalation. For example, a previously published meta-analysis revealed that there is a proportional relationship between higher drug exposure and increased probability of response. In addition, sub-therapeutic blood levels rather than true resistance to therapy with TKIs have been accounted as reasons for disease progression [7]. Furthermore, upfront administration of higher doses given intermittently to mitigate toxicities, under individualized pharmacokinetic guidance, was shown to exhibit improved efficacy for a variety of TKIs, while escalation to a higher dose at the time of progression resulted in more than 5 months added progression-free survival period for patients with renal cell cancer receiving sunitinib. The latter data suggest that sunitinib dose escalation is a valid strategy to overcome the initial development of acquired resistance and resensitize the tumors to the drug, though still transiently [8].

To test our hypothesis on refining the clinical use of TKIs by optimization of treatment strategies using a chemotherapy-like schedule of pulsatile, high doses, we evaluated in vitro the antitumor efficacy of short exposure to a high concentration of the antiangiogenic TKI, sunitinib. We found that a single exposure to 20 µM sunitinib for 6–9 h resulted in complete inhibition of tumor cell growth. In addition, repeated exposure of tumor cells to pulses of high concentrations of sunitinib remained efficacious and did not induce resistance. Furthermore, pulsatile treatment with high-dose sunitinib of tumors growing on the chorioallantoic membrane (CAM) of the chicken embryo significantly impaired tumor growth [9]. These results prompted the initiation of a phase I clinical trial, where once weekly administration of 300 mg of sunitinib was shown to be feasible, with tolerability similar to the standard daily administration of 50 mg. Promising prolonged disease stabilization in a heavily pretreated group of patients was observed (Rovithi et al., unpublished data), and further results are eagerly awaited. For other TKIs, these short time, high-dose exposures also reveal impressive antitumor activities in vitro, for example with the antiangiogenic TKIs regorafenib and sorafenib (Rovithi et al., unpublished data). Therefore, a second study with high dose once weekly sorafenib has been recently started in our institution as well (ClinicalTrials.gov Identifier: NCT02636426).

In summary, there are several approaches taken to uncover and exploit the full potential of antiangiogenic TKIs. Common one is the development of combinational strategies with (a) inhibitors of the escape, prosurvival, parallel to angiogenesis pathways in addition to the primary target, (b) chemotherapy or (c) immune checkpoint inhibitors following the hypothesis that blunting the function of VEGF would reverse all the VEGF-mediated immunosuppressive effects and augment their efficacy. Major concern for every combinational approach remains the development of serious toxic side effects. In parallel, identification of optimal treatment scheduling and dosing is actively pursued by us and others and the first results seem promising when these antiangiogenic TKIs are used in a pulsatile, high-dose schedule, similar to the use of classical chemotherapy. Eleven years after the first FDA approval of an antiangiogenic TKI, sorafenib for renal cell cancer, the landscape of these small molecules faces multiple challenges regarding their repositioning in the ever-evolving cancer treatment armamentarium. Perhaps the best is yet to come.
